# Micro-Engineered Smart ZnO Inverse Opal Electrodes with AgCuS Nanocrystal Amplification for Ultrasensitive Anti-LGI1 Antibody Detections

**DOI:** 10.3390/mi17080935

**Published:** 2026-08-06

**Authors:** Dong Li, Hao Wu, Lina Wang, Peng Yu, Langping Tu, Hongfei Li, Le Liu, Long Shao

**Affiliations:** 1 School of Materials Science and Engineering, Changchun University of Science and Technology, Changchun 130000, China; lidong@mails.cust.edu.cn (D.L.); wangln@cust.edu.cn (L.W.); yupeng2006@mails.cust.edu.cn (P.Y.); tulangping@cust.edu.cn (L.T.); hongfeili@cust.edu.cn (H.L.); 2 State Key Laboratory of Intelligent Manufacturing Equipment and Technology, Huazhong University of Science and Technology, Wuhan 430074, China; moxingkong@hust.edu.cn; 3 Research Center for Advanced Electronics Manufacturing, Huazhong University of Science and Technology, Wuhan 430074, China; 4 Flexible Electronics Research Center, Huazhong University of Science and Technology, Wuhan 430074, China; 5 College of Mathematics and Physics, Henan University of Urban Construction, Pingdingshan 467036, China; leliu@huuc.edu.cn

**Keywords:** smart electrode, inverse opal, AgCuS nanocrystal, photoelectrochemical biosensor, intelligent diagnostic platforms

## Abstract

Micro-fabrication and structural engineering of an ultrasensitive photoelectrochemical (PEC) micro-sensing platform have been developed, based on ZnO inverse opal electrodes with signal amplification enhanced by AgCuS nanocrystals, specifically designed for the detection of anti-LGI1 antibodies. The delayed light effect inherent in the ZnO inverse opal structure enhances photoelectrochemical efficiency by extending the effective optical path. Incorporation of AgCuS nanocrystals significantly augments the photoelectric sensitivity of the ZnO inverse opal, maximizing visible light utilization, accelerating charge transfer kinetics, and substantially enhancing photocurrent generation. Leveraging the uniform porous architecture of the ZnO inverse opal, the ZnO/AgCuS film provides an expansive surface area for biomolecule immobilization and facilitates enhanced electron transport. The ZnO/AgCuS heterogeneous film was innovatively implemented as a PEC bioassay platform. Under optimized conditions, the biosensor exhibited a linear detection range of 0.01–500 ng/mL and a detection limit of 13 pg/mL for Anti-LGI1. Furthermore, the fabricated PEC biosensor demonstrated robust performance in human serum sample analyses, characterized by high repeatability, long-term stability, and excellent specificity. This work proposes a micro-manufacturing strategy for high-performance PEC biodevices, promoting the development of intelligent diagnostic platforms for autoimmune encephalitis.

## 1. Introduction

Autoimmune encephalitis (AE) refers to a group of brain inflammations mediated by autoimmune mechanisms, characterized by the production of a series of autoantibodies acting against the surface proteins or synaptic proteins of neurons [1]. Its annual incidence rate is approximately 5–8 per 100,000 [1]. Among these cases, anti-leucine-rich glioma inactivated protein 1 (LGI1) antibody-related encephalitis is the second most common AE mediated by anti-neuronal surface protein antibodies, after anti-N-methyl-D-aspartate receptor (NMDAR) antibody-related encephalitis, which has a relatively high recurrence rate and a mortality rate of up to 6% [2]. The recurrence rate of anti-LGI1-related encephalitis is high [3], and it leads to significant cognitive impairment [4]. Even after immunotherapy, approximately 65% of patients still have residual symptoms and are unable to live independently or return to work or study [5,6], imposing a heavy burden on their families and society. The diagnosis of anti-LGI1-related encephalitis depends on the detection of anti-LGI1 antibodies. Therefore, developing highly sensitive and highly specific detection methods for anti-LGI1 antibody is of crucial importance. Hence, several techniques for detecting anti-LGI1 antibody have been developed, including enzyme-linked immunosorbent assays and cell-based assays [7]. Despite their high sensitivity, these approaches usually require complex procedures, high costs, and lengthy processing periods. Therefore, there is a need for a novel, rapid, and highly sensitive anti-LGI1 antibody detection method to efficiently diagnose anti-LGI1-related encephalitis and reduce its mortality rate.

Photoelectrochemical (PEC) biosensors have been established as state-of-the-art analytical systems for biological detection, distinguished by exceptional biomolecular affinity and inherent sensitivity that surpass conventional methodologies [8,9,10]. For example, Wang and his team engineered a novel label-free photoelectrochemical biosensor utilizing BiPO_4_/TiO_2_ heterostructured films for prostate-specific antigen (PSA) detection [11]. The resultant PEC biosensor demonstrated excellent specificity, reproducibility, long-term stability, and notable analytical performance in human serum [9,10,12,13]. This detection method operates via the efficiency of photoactive semiconductor materials in converting light energy to electrical energy, facilitating the measurement of target biomarkers. During photoelectrochemical detection, photosensitive compounds are excited by precisely controlled light wavelengths, initiating electron transfer processes that generate measurable electrical signals. Consequently, photoelectrochemical detection technology achieves high analytical sensitivity, rendering it particularly effective for quantifying trace biomolecules and chemical analytes. The photocurrent, arising from photoelectric conversion within photoelectrochemical biosensing platforms, constitutes the primary quantitative output signal enabling rapid biomarker quantification [14,15]. PEC biosensors are categorized based on their photosensitive constituents into inorganic [16,17,18], organic [19], or composite materials [20,21]. Representative inorganic photosensitive materials include ZnO [22,23,24], TiO_2_ [25,26,27,28], and reduced graphene oxide [29,30,31]. ZnO serves as a prominent photocatalyst owing to its exceptional electrical conductivity, efficient charge transfer capabilities, biocompatibility, and robust chemical stability. However, ZnO’s wide bandgap (≈3.37 eV) confines light absorption predominantly to the ultraviolet region near 380 nm, thereby limiting optimal utilization of the solar spectrum [32,33,34,35]. Furthermore, ultraviolet irradiation exhibits pronounced oxidative capacity, which can induce biomolecular degradation [36]. Consequently, a principal challenge for ZnO-based photoelectrodes lies in achieving efficient visible light harvesting; the formation of heterostructures through strategic material hybridization has proven effective in enhancing the photoelectric response to mitigate this limitation [37,38].

Heterojunction interfaces in semiconductors enhance optoelectronic efficiency by suppressing the recombination of photogenerated charge carriers and broadening the spectral range of light absorption [39]. Extensive investigations demonstrate that surface modification of ZnO with narrow-bandgap semiconductors, such as CdS, CdSe, CdTe, and PbS, significantly improves PEC performance [37,40,41].

However, the inherent toxicity of heavy metals within these materials constrains their commercial viability. AgCuS, an environmentally benign semiconductor, has garnered significant research attention due to its high visible light absorption coefficient and photostability in photovoltaic applications. Recent advances in AgCuS-based PEC biosensing have demonstrated its great potential for biomarker detection: Li et al. developed an AgCuS/g-C_3_N_4_ heterostructure for cardiac troponin I detection with a LOD of 2.3 pg/mL, while Zhang et al. reported an AgCuS/TiO_2_ nanotube array sensor for alpha-fetoprotein detection with excellent stability over 30 days. Despite these promising results, no previous studies have combined a ZnO inverse opal structure with AgCuS nanocrystals for anti-LGI1 detection, which defines the unique novelty of our work in addressing the current research gap of low-sensitivity, time-consuming anti-LGI1 detection methods [42,43,44]. Furthermore, AgCuS exhibits exceptional chemical stability and biocompatibility as an inorganic semiconductor, establishing it as a highly promising candidate for substrate-based PEC biosensing applications [45].

**Scheme 1:**
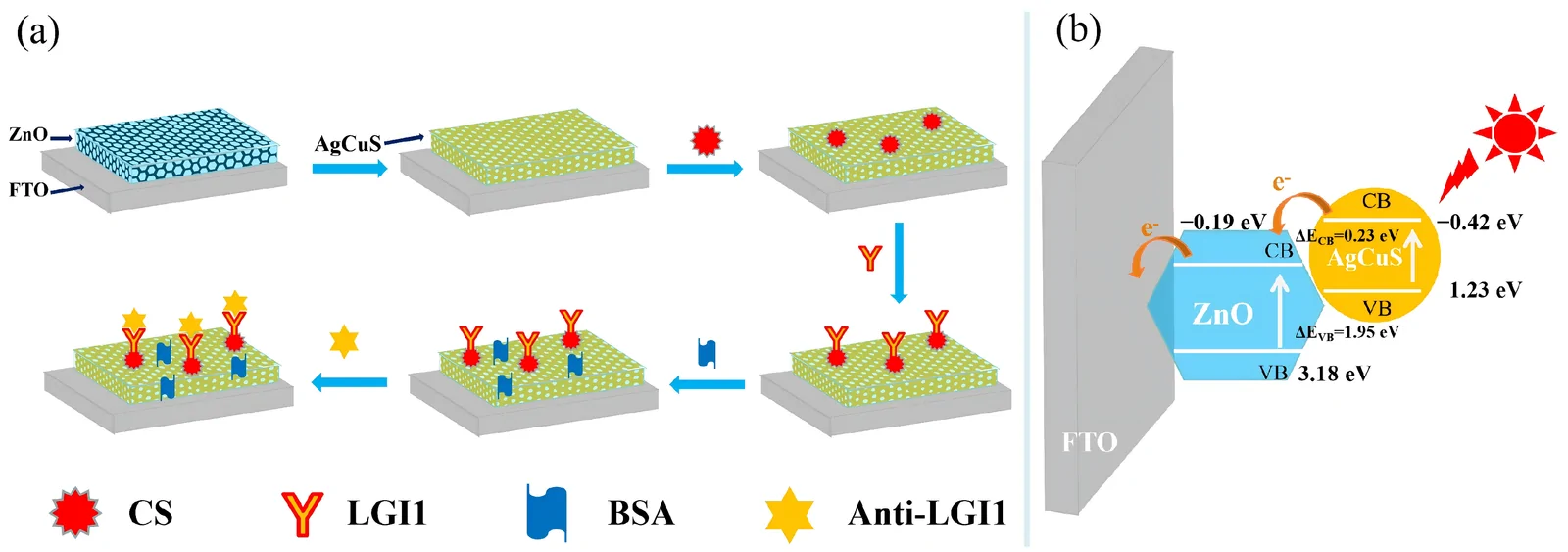
(**a**) The fabrication procedure of the ZnO/AgCuS photoelectrochemical biosensor; (**b**) the operational mechanism of this biosensor. VB and CB represent the valence band and conduction band, respectively.

Herein, we report on the development of an innovative ZnO/AgCuS heterostructured film-based label-free PEC biosensor for anti-LGI1 antibody detection (Scheme 1). The ZnO layer employs a three-dimensional inverse opal (IO) photonic crystal structure, which enhances photochemical processes and the photovoltaic response of solar cells through an increase in the effective optical path length [46,47]. Furthermore, the uniform porous framework and extensive specific surface area of the ZnO IO matrix shorten the diffusion distance between target analytes and the fluorine-doped tin oxide (FTO) substrate. This structural configuration facilitates efficient electron transfer and supports biomolecule immobilization [46,48]. Energy level matching between ZnO IO and AgCuS nanocrystals (NCs) further enables the ZnO/AgCuS heterostructure to efficiently harvest visible light, promoting charge carrier separation, markedly augmenting photocurrent response, and alleviating UV-induced protein damage. Despite the lack of prior exploration regarding ZnO/AgCuS heterostructures in PEC.

## 2. Methods

### 2.1. Preparation of ZnO IO

Uniformly monodisperse poly (methyl methacrylate) (PMMA) spheres of controlled dimensions were synthesized via an established emulsion polymerization protocol. Vertical deposition self-assembly was subsequently employed to fabricate the PMMA colloidal crystal template. The resultant PMMA colloidal suspension was deposited onto a hydrophilic, FTO-coated glass substrate and aged at 30 °C for 36 h. Self-assembly of PMMA colloidal spheres into a highly ordered array on the FTO substrate was mediated by solvent evaporation-induced capillary forces. To enhance mechanical stability, the PMMA template was sintered at 130 °C for 30 min. A zinc-ion precursor solution was prepared by dissolving zinc nitrate hexahydrate (Zn(NO_3_)_2_·6H_2_O) in absolute ethanol. Stoichiometric quantities of tetraethyl orthosilicate (TEOS) and citric acid were introduced as chelating agents, followed by continuous stirring until a homogeneous colorless solution formed. This precursor solution was then infiltrated into the PMMA template interstices via capillary action at ambient temperature. Fabrication of the ZnO IO structure was achieved by calcining the infiltrated samples at 450 °C for 4 h, thereby removing the PMMA template via thermal decomposition.

### 2.2. Preparation and Modification of AgCuS NCs

In a standard synthesis protocol, thioacetamide (TAA, 0.4 mmol) and oleylamine (OLA, 4.0 mL) were combined in a 50 mL three-neck flask. The mixture was heated to 200 °C under a nitrogen atmosphere and maintained at this temperature for 15 min to yield a transparent solution. Concurrently, within an inert-atmosphere glovebox, a solution of silver nitrate (AgNO_3_, 0.2 mmol) dissolved in OLA (2.0 mL) was prepared and promptly injected into the flask. The clear solution immediately turned dark brown, indicative of Ag_2_S nanocrystal nucleation. Similarly, a solution of copper (II) chloride (CuCl_2_, 0.2 mmol) in OLA (2.0 mL), prepared within the glovebox, was rapidly injected into the reaction vessel. Subsequently, the mixture was heated to 220 °C and maintained at this temperature for 5 min. The resulting nanocrystals underwent repeated purification cycles via precipitation using chloroform and acetone as the solvent/anti-solvent pair, and were ultimately redispersed in chloroform for storage and characterization.

### 2.3. Preparation of FTO/ZnO/AgCuS Electrode

The FTO/ZnO electrode was modified by spin-coating with 100 μL of a 10 mL toluene solution containing dispersed AgCuS NCs. Subsequently, the electrode was annealed at 80 °C for 2 h, dried under ambient conditions, and then rinsed with PBS.

All materials used in the experiment could be viewed in the Supplementary Materials.

### 2.4. PEC Measurements

The FTO working electrode surface was initially modified with a ZnO/AgCuS heterogeneous layer. Subsequently, 10 μL of 0.05 wt% chitosan (CS) solution was deposited onto the surface and air-dried at ambient temperature for 1 h. The resulting FTO/ZnO/AgCuS electrode was then extensively rinsed with NaOH solution and deionized water. Following a 1 h drying period at room temperature, 50 μL of 5% glutaric dialdehyde aqueous solution was applied to activate the chitosan. The electrode was thoroughly rinsed with deionized water. Next, the surface was functionalized through deposition of 50 μL of 20 μg/mL LGI1, followed by incubation at 4 °C for over 15 h. Unbound LGI1 was removed by PBS washing. A 50 μL solution of 1% bovine serum albumin (BSA) was then deposited onto the electrode and incubated at 37 °C for 1 h to block nonspecific binding sites. After extensive PBS rinsing, 50 μL solutions of Anti-LGI1 at varying concentrations were deposited onto the modified electrode. Incubation proceeded at 37 °C for 1.5 h, followed by rigorous PBS washing, yielding the final PEC biosensor for subsequent experimentation.

Electrochemical and photoelectrochemical analyses were conducted utilizing a standard three-electrode configuration. A saturated calomel electrode (SCE) served as the reference electrode, while a platinum wire functioned as the counter electrode. The working electrode comprised a modified FTO substrate with a defined surface area of 1.0 ± 0.1 cm^2^. Photocurrent measurements were performed at a constant applied potential of 0.6 V (vs. SCE) under illumination from a 500 W xenon lamp. The electrolyte consisted of phosphate-buffered saline (PBS) solution (pH 7.2) containing 0.06 M ascorbic acid (AA). The experimental setup for photoelectrochemical characterization is schematically illustrated in Scheme S1 and all apparatus used in the experiment can be viewed in the Supplementary Materials.

## 3. Results and Discussion

### 3.1. Characterization

**Figure 1:**
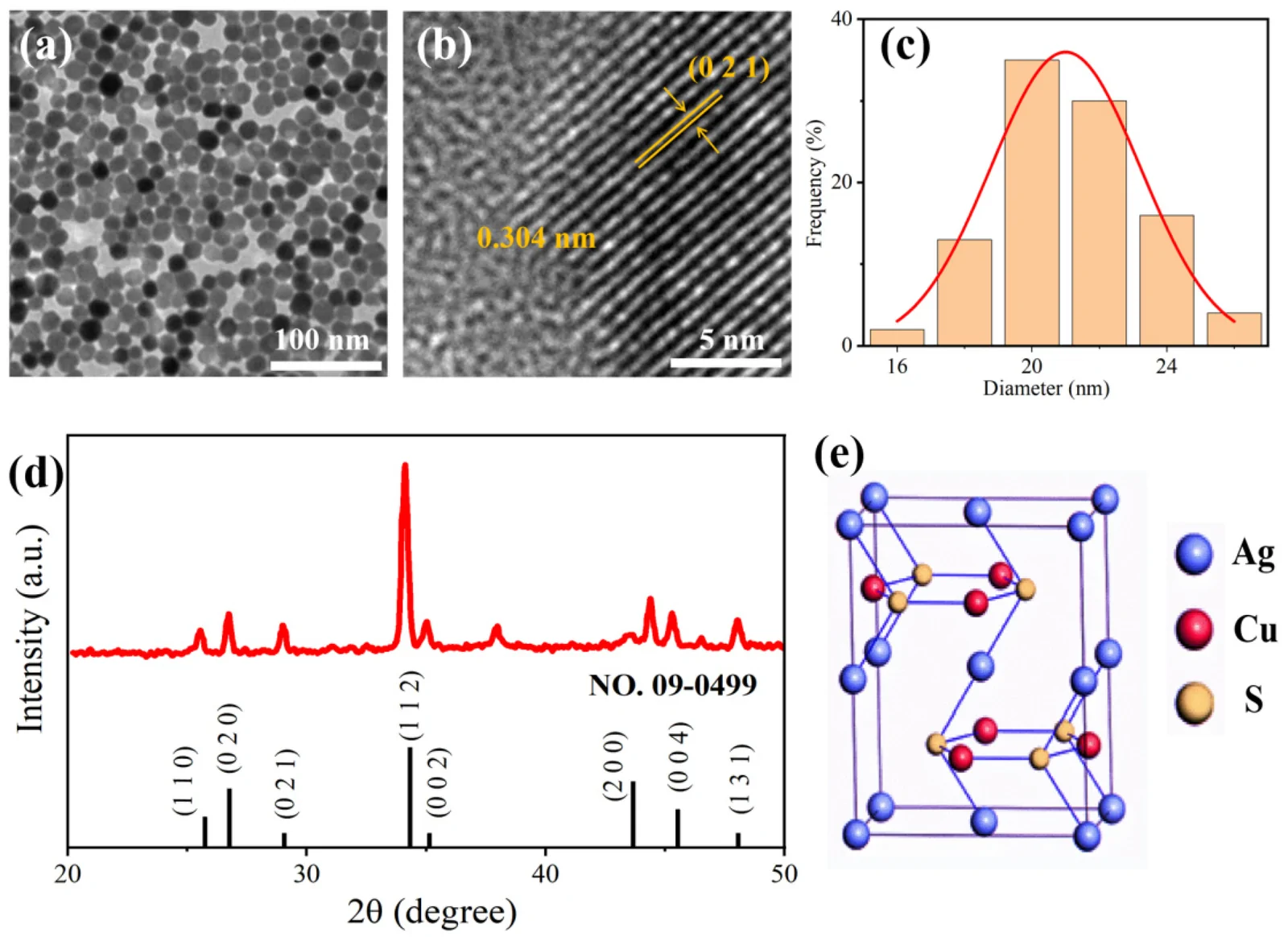
(**a**) TEM of the AgCuS NCs, (**b**) HRTEM of the AgCuS NCs, (**c**) particle size statistics, (**d**) XRD pattern, (**e**) schematic of the crystal structure of the AgCuS NCs.

Transmission electron microscopy (TEM) analysis confirmed the successful synthesis of AgCuS NCs. Figure 1a reveals a highly ordered arrangement of NCs on the TEM, exhibiting high homogeneity with an average particle size of approximately 21 nm. High-resolution TEM (HRTEM) imaging (Figure 1b) resolved lattice fringes corresponding to the (021) planes, exhibiting a d-spacing of 3.04 Å. The particle size distribution (Figure 1c) conforms to a Gaussian profile, with the majority (>90%) of particles confined to a narrow range of 16–26 nm, confirming their monodisperse nature. X-ray diffraction (XRD) characterized the phase purity of the AgCuS NCs. Rietveld refinement performed using TOPAS 4.2 software yielded an excellent fit to the experimental diffractogram (Figure 1d). All diffraction peaks index to a tetragonal unit cell, matching the standard reference pattern for AgCuS (JCPDS No. 09-0499). The absence of extraneous peaks and the presence of sharp diffraction signals indicate high crystallinity. Rietveld analysis definitively confirmed the tetragonal phase with space group Cmc2_1_ for the synthesized AgCuS NCs (Figure 1e).

**Figure 2:**
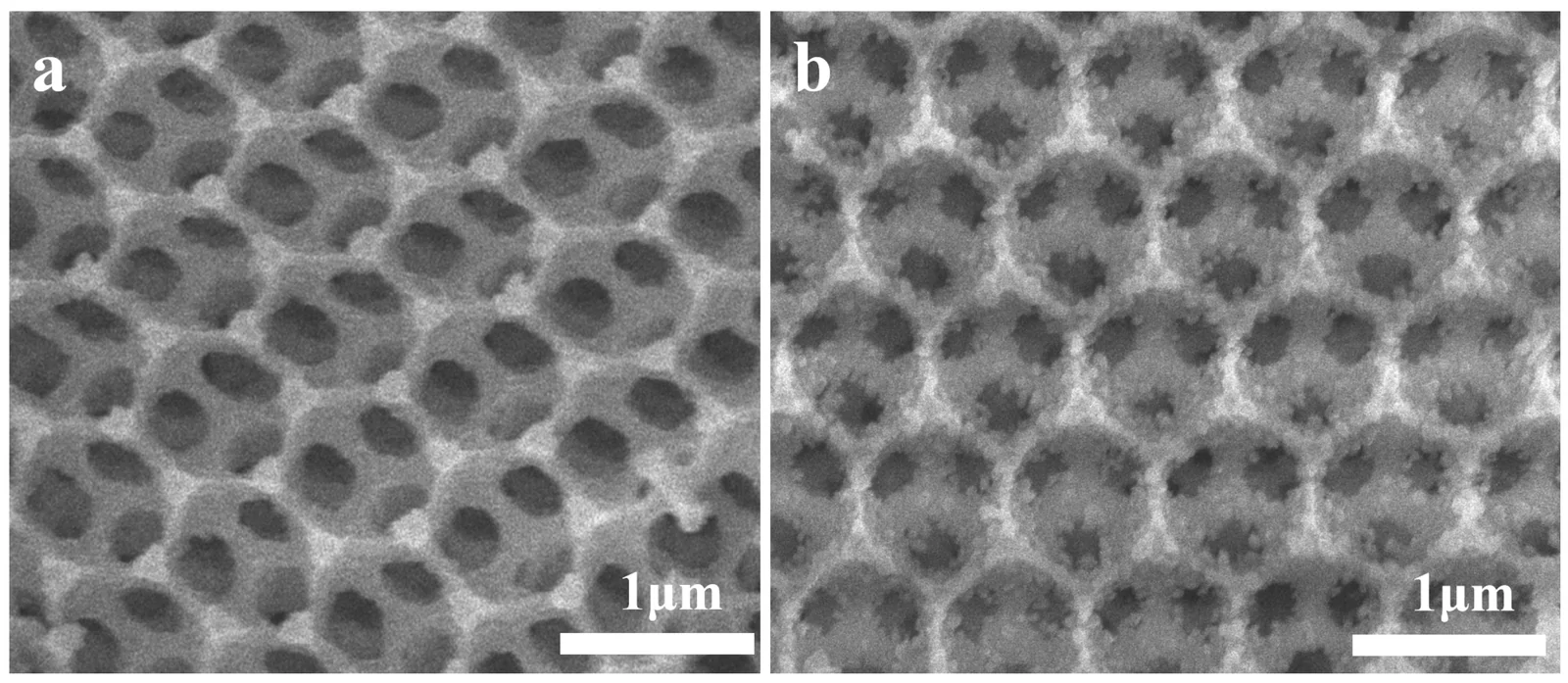
(**a**) SEM image of the FTO/ZnO thin films, (**b**) SEM image of the FTO/ZnO/AgCuS thin films.

Figure 2a presents a field-emission scanning electron microscopy (SEM) image of the ZnO IO thin film, revealing a well-defined three-dimensional pore structure with pore diameters of approximately 750 nm. Correspondingly, the SEM image of the AgCuS NCs modified ZnO IO thin film (Figure 2b) exhibits numerous synthesized AgCuS NCs adhered to both the external and internal surfaces of the ZnO IO framework. This modification substantially augments the specific surface area of the electrode, thereby significantly enhancing surface biological protein adsorption and consequently improving the sensor’s detection performance.

**Figure 3:**
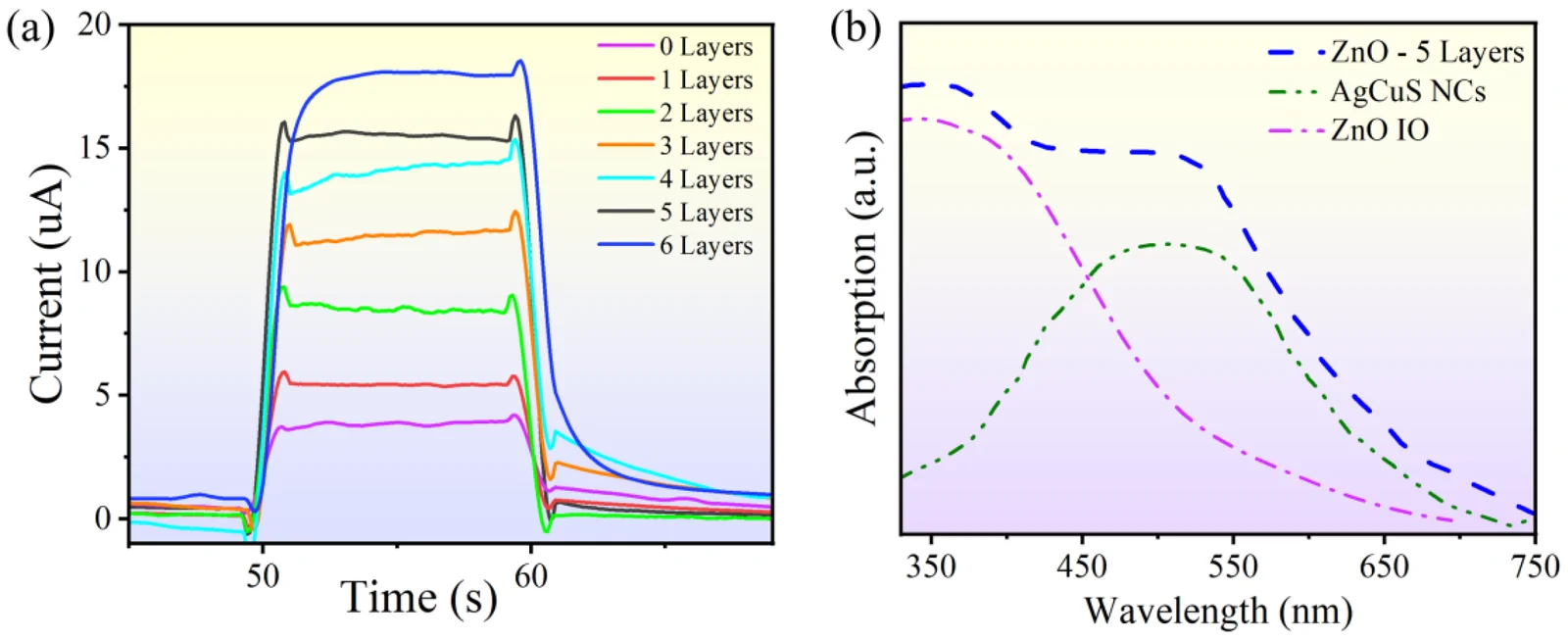
(**a**) Photocurrent measurements for ZnO/AgCuS composite electrodes with varying numbers of spin-coated AgCuS nanocrystal layers. (**b**) Absorption spectra of ZnO inverse opals, AgCuS nanocrystals, and composite electrodes coated with 5 layers of AgCuS nanocrystals.

Compared to the FTO/ZnO film, the FTO/ZnO/AgCuS heterogeneous film demonstrated enhanced photocurrent response, as depicted in Figure 3a. However, the composite electrode exhibited diminished photocurrent when the number of spin-coated AgCuS NCs layers exceeded five layers. This reduction is attributed to excessive AgCuS NCs deposition on the electrode surface, which elevates diffusion resistance and establishes additional recombination centers.

Additionally, we investigated the spectral response characteristics of the ZnO/AgCuS heterostructured film. As illustrated in Figure 3b, ZnO IO exhibits primary absorption within the ultraviolet region, whereas AgCuS NCs absorb visible light. Consequently, the ZnO/AgCuS heterostructured film demonstrated absorption across both the ultraviolet and visible spectral ranges. Significantly, this composite heterostructured film exhibited significantly enhanced visible light absorption compared to the ZnO IO film alone, thus indicating improved photoelectric conversion efficiency in the material.

### 3.2. Characterization of the Photoelectrochemical Biosensor Construction Process

**Figure 4:**
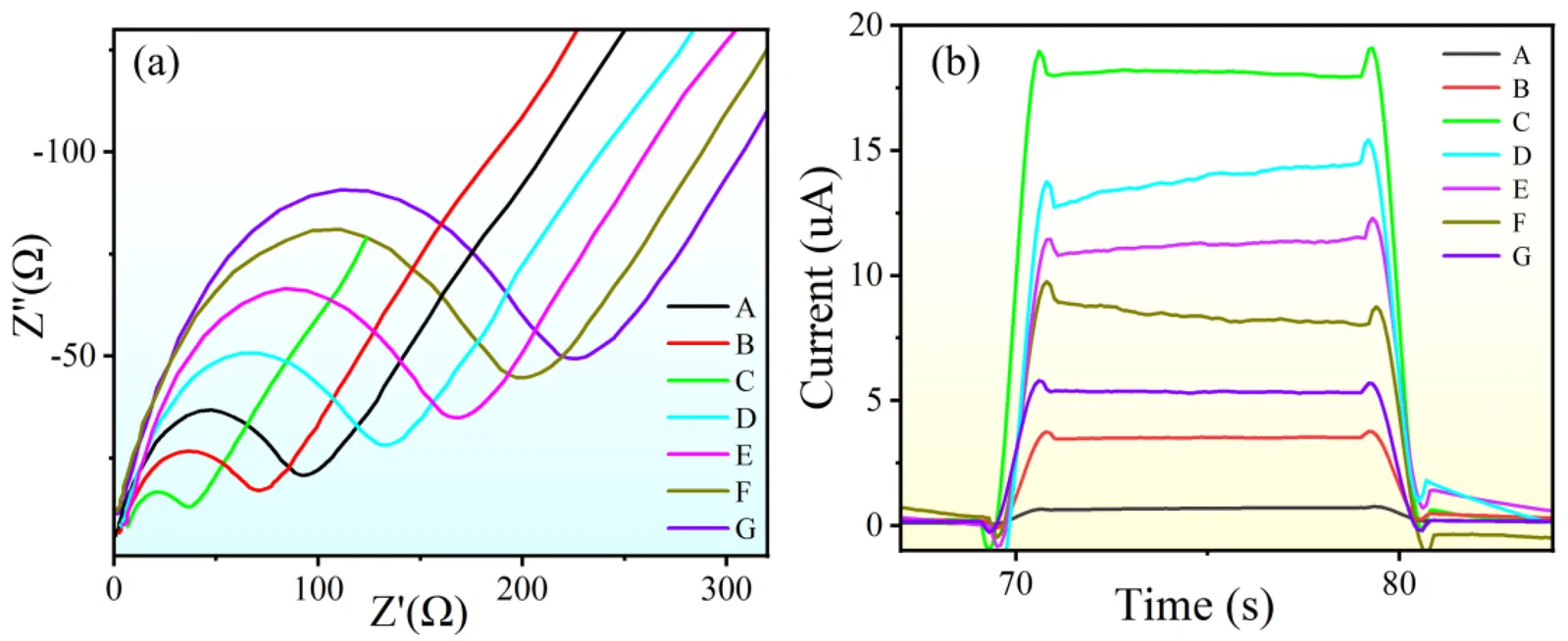
(**a**) EIS profile and (**b**) photocurrent response at each step of electrode modification: (A) FTO, (B) FTO/ZnO, (C) FTO/ZnO/AgCuS, (D) FTO/ZnO/AgCuS/CS, (E) FTO/ZnO/AgCuS/CS/LGI1, (F) FTO/ZnO/AgCuS/CS/LGI1/BSA, (G) FTO/ZnO/AgCuS/CS/LGI1/BSA/Anti-LGI1.

The assembly process of the biosensor electrode was monitored employing electrochemical impedance spectroscopy (EIS) and photocurrent response measurements (Figure 4). EIS, a standard technique for characterizing electrode modification processes, provided electron transfer resistance (*R*_et_) values. *R*_et_ data were acquired in a 0.01 M phosphate-buffered saline (PBS) solution (pH 7.2), comprising 0.1 M KCl and an equimolar mixture of 0.05 M K_3_[Fe(CN)_6_]/K_4_[Fe(CN)_6_], under open circuit potential conditions (0.6 V) over a frequency range of 0.01 to 100 Hz, with *R*_et_ represented by the semicircle diameter in Nyquist plots. As illustrated in Figure 4a, the ZnO IO exhibited high conductivity, resulting in a significant decrease in electrode resistance. The FTO/ZnO/AgCuS electrode displayed a smaller *R*_et_ value than the FTO/ZnO electrode. Subsequent chitosan (CS) coating substantially elevated resistance owing to its inherent insulating properties. Further modifications with LGI1 (50 μL, 20 μg/mL) and bovine serum albumin (BSA; 50 μL, 1%) induced slight increases in resistance, attributable to electron transfer hindrance by these organic molecules. The introduction of 50 μL of 10 ng/mL Anti-LGI1 induced significant alterations in the impedance spectrum, indicative of surface binding events. This methodology successfully yielded a label-free PEC biosensor based on a ZnO/AgCuS heterostructure. The performance of the PEC biosensor was evaluated at a fixed bias potential of 0.6 V under illumination from a 500 W xenon lamp in PBS solution (pH 7.2) containing 0.06 M ascorbic acid (AA) as an electron donor.

Figure 4b presents the photocurrent response variations throughout the electrode fabrication sequence, corroborating the successful assembly of the PEC biosensor through these observed alterations. The results indicate a significantly enhanced photocurrent for the FTO/ZnO electrode compared to bare FTO, attributed to the superior conductivity and photoelectric conversion efficiency of AgCuS NCs and the successful integration of the ZnO IO structure. The introduction of the ZnO/AgCuS heterojunction film amplified the photocurrent response by approximately sixfold, highlighting its critical role in augmenting the biosensor’s photoelectric efficiency. This enhancement is predominantly ascribed to the favorable energy band alignment between ZnO IO and AgCuS NCs, enabling more efficient injection of excited-state electrons into the ZnO IO layer. This synergistic mechanism facilitates charge transfer, minimizes carrier recombination, and substantially enhances photocurrent generation. Furthermore, the slow light effect inherent to the ZnO IO structure extends the photovoltaic response duration and effective optical path length, potentially improving light harvesting and conversion efficiency. Subsequent modification of the electrode surface with insulating organic CS, LGI1, BSA, and Anti-LGI1-rogressively attenuated the photocurrent, as these layers impeded electron transfer kinetics and diminished current sensitivity. The sequential photocurrent shifts observed across the modification stages corroborate the successful fabrication of a label-free PEC biosensor utilizing the ZnO/AgCuS heterojunction film.

### 3.3. Optimization of the Detection Conditions

**Figure 5:**
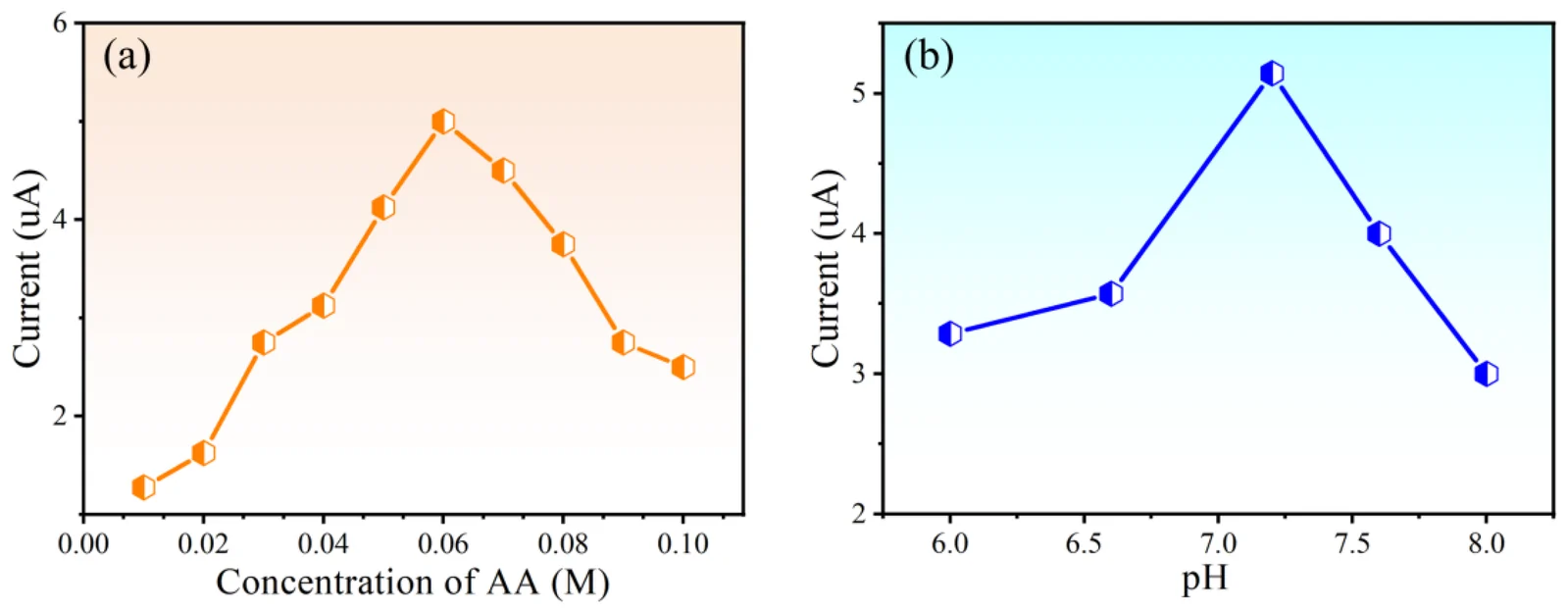
Current (**a**) with different AA concentrations and (**b**) at various pH levels (6.0–8.0). The concentrations of LGI1 and Anti-LGI1 are 20 μg/mL and 10 ng/mL, respectively.

To develop a highly effective PEC biosensor, we investigated the optimization of AA concentration and solution pH. Within the test solution, AA serves as an electron donor, facilitating the generation of a stable and substantial photocurrent signal while simultaneously suppressing electron-hole recombination. Consequently, AA concentration was evaluated as a critical parameter. As depicted in Figure 5a, photocurrent intensity increases progressively with AA concentrations from 0.02 M to 0.06 M, but declines between 0.06 M and 0.10 M. This trend indicates that 0.06 M AA provides sufficient electrons for photoinduced holes, achieving saturated electron donation. Furthermore, solution pH modulates the photoelectric response of the electrode by altering the activity of surface biomolecules. For detection, a pH of 7.2 was identified as optimal, yielding the maximum photocurrent, as demonstrated in Figure 5b.

### 3.4. Photoelectrochemical Detection of Anti-LGI1

**Figure 6:**
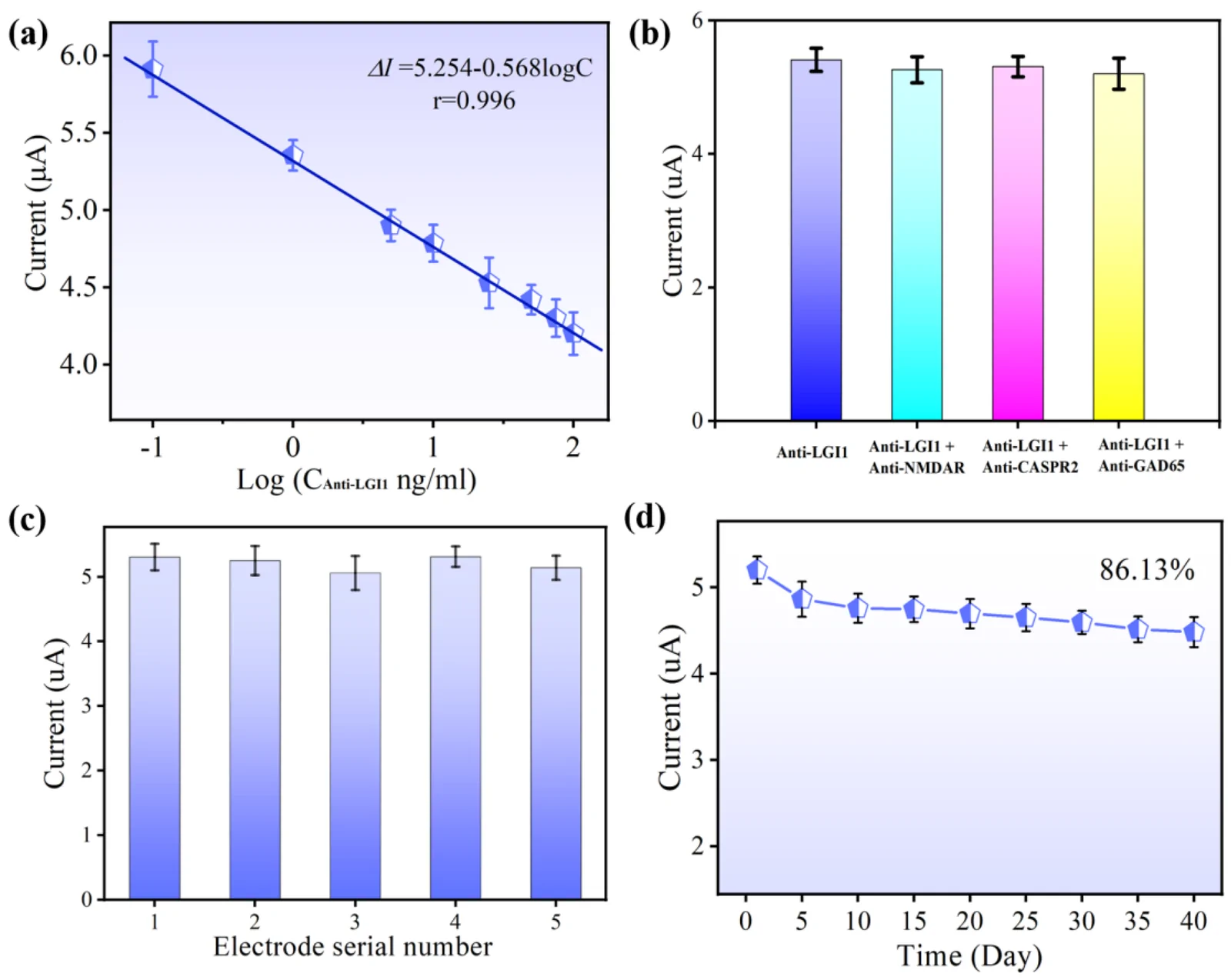
(**a**) Calibration curves demonstrating the photocurrent response of the ZnO/AgCuS sensor across various Anti-LGI1 concentrations. (**b**) Specificity analysis using the target anti-LGI1 (10 ng/mL) and other interferents (100 ng/mL). (**c**) Assessment of the photocurrent response repeatability for the sensor. (**d**) Long-term stability evaluation of the sensor over a 40-day period.

Under optimal conditions, variations in Anti-LGI1 concentration (50 μL) were quantified by monitoring photocurrent shifts using the developed PEC biosensing platform. Figure 6a depicts the calibration curve correlating photocurrent response with Anti-LGI1 concentration. A logarithmic decrease in photocurrent was observed across the 0.01–500 ng/mL Anti-LGI1 range. This relationship is described by the regression equation *ΔI* = 5.254 − 0.568 log C_Anti-LGI1_ (R^2^ = 0.996), where *ΔI* represents the photocurrent of the FTO/ZnO/AgCuS/CS/LGI1/BSA electrode following incubation with 50 μL Anti-LGI1 solutions. Increasing Anti-LGI1 concentrations impeded electron transfer from AA to the PEC sensor, resulting in a progressive reduction in photocurrent intensity. A detection limit of 13 pg/mL was determined at a signal-to-noise ratio of 3 (S/N = 3). Compared to heterostructure photo-electrochemical biosensors employing alternative materials, this PEC biosensor exhibits a wider linear range and a lower detection limit for Anti-LGI1 quantification.

To evaluate the relative standard deviation (RSD), Anti-LGI1 standard solutions at varying concentrations were spiked into a standard human serum sample. As presented in Table S1, analytical recoveries ranged from 96% to 105%, with RSD values less than 5.6%. These results confirm the high sensitivity and precision of the ZnO/AgCuS heterostructured film-based label-free PEC biosensor for detecting Anti-LGI1 in authentic biological matrices.

### 3.5. Specificity, Reproducibility, and Stability of the PEC Biosensor

The selectivity of the engineered Anti-LGI1 biosensor was evaluated by introducing interfering agents, including Anti-NMDAR, Anti-CASPR2, and Anti-GAD65. Exposure of electrodes to mixtures containing 100 ng/mL interfering proteins and 10 ng/mL Anti-LGI1 elicited photocurrent responses arising from specific binding interactions (Figure 6b). These signals remained stable without significant variations, indicating that nonspecific adsorption effects were minimal. Consequently, this supports that the label-free PEC biosensor employing ZnO/AgCuS heterogeneous films demonstrated preliminary analytical selectivity that is favored for future studies in complex clinical samples.

To assess the photoelectric response to 10 ng/mL Anti-LGI1, five PEC biosensors were fabricated under identical conditions. As illustrated in Figure 6c, photocurrent intensity exhibited negligible variation across these biosensors, confirming the high reproducibility of the ZnO/AgCuS heterogeneous film-based label-free PEC biosensor. Figure 6d demonstrates the long-term stability of a ZnO/AgCuS heterogeneous film electrode stored at 4 °C. The photoelectric response was measured every five days using 10 ng/mL Anti-LGI1. After 40 days, the electrode retained 86.13% of its initial performance, with photocurrent exhibiting a gradual decline over time, highlighting the robust long-term stability of the ZnO/AgCuS heterogeneous film-based label-free PEC biosensor.

## 4. Conclusions

This research describes the advancement of dependable PEC biosensors that feature substantially lowered background noise and heightened sensitivity, accomplished via heterojunction engineering. The heterojunction configuration curbs the recombination of photogenerated electron-hole pairs, thereby augmenting semiconductor photoelectric efficiency while greatly expanding the light absorption spectrum. By utilizing Anti-LGI1 as a model analyte, we comprehensively evaluated the analytical efficacy of the engineered PEC biosensors. A ZnO/AgCuS heterojunction film with a uniform porous nanostructure and ample surface area was developed to enable efficient electron transfer and biomolecule immobilization, yielding significant amplification of photocurrent responses. Under refined conditions, the biosensor displayed a wide linear detection span from 0.01 to 500 ng/mL, with a calculated limit of detection at 13 pg/mL. Moreover, the biosensor exhibited superior performance in human serum samples, showing exceptional reproducibility, outstanding long-term stability, and high specificity (the performance comparison of commercial anti-LGI1 detection methods and the developed PEC biosensor is shown in Table S3). In the future, we will further optimize the preparation process to achieve one-step functionalization, thereby reducing the manufacturing costs and time required for clinical application. These efforts will further advance the practical utilization of this PEC biosensor in the clinical diagnosis of autoimmune encephalitis.

## Data Availability

The data presented in this study are available on request from the corresponding author.

## Supplementary Materials

The following supporting information can be downloaded at: https://www.mdpi.com/article/10.3390/mi17080935/s1. Detailed information of materials and apparatus; Scheme S1: The photoelectrochemical measurement process; Table S1: Detection of Anti-LGI1 in human standard serum; Table S2: Performance comparison of commercial anti-LGI1 or LGI1 detection methods and the developed PEC biosensor; Table S3: Synthesis parameter gradient screening results and optimized values.
