# Supplementary material for: Micro-Engineered Smart ZnO Inverse Opal Electrodes with AgCuS Nanocrystal Amplification for Ultrasensitive Anti-LGI1 Antibody Detections
Source: Micromachines (Basel). 2026 Aug 6;17(8):935. doi: 10.3390/mi17080935 (PMC13515716; doi:10.3390/mi17080935)
Supplement: Supplementary file 1 [file micromachines-17-00935-s001.zip › micromachines-4463012-supplementary.pdf]

Article

Micro-Engineered Smart ZnO Inverse Opal Electrodes with AgCuS Nanocrystal Amplification for Ultrasensitive Anti-LGI1 Antibody Detections

Dong Li [1,#], Hao Wu [2,3,4,#], Lina Wang [1], Peng Yu [1], Langping Tu [1], Hongfei Li [1], Le Liu [5], Long Shao [1,*]

1 School of Materials Science and Engineering, Changchun University of Science and Technology, Changchun 130000, China; lidong@mails.cust.edu.cn (D.L.); wangln@cust.edu.cn (L.W.); yupeng2006@mails.cust.edu.cn (P.Y.); tulangping@cust.edu.cn (L.T.); hongfeili@cust.edu.cn (H.L.); shaolong@cust.edu.cn (L.S.)

2 State Key Laboratory of Intelligent Manufacturing Equipment and Technology, Huazhong University of Science and Technology, Wuhan 430074, China; moxingkong@hust.edu.cn (H.W.)

3 Research Center for Advanced Electronics Manufacturing, Huazhong University of Science and Technology, Wuhan 430074, China; moxingkong@hust.edu.cn (H.W.)

4 Flexible Electronics Research Center, Huazhong University of Science and Technology, Wuhan 430074, China; moxingkong@hust.edu.cn (H.W.)

5 College of Mathematics and Physics, Henan University of Urban Construction, Pingdingshan 467036, China; leliu@huuc.edu.cn (L.L.)

# These authors contributed equally to this work.

* Correspondence: shaolong@cust.edu.cn (L.S.)

Materials

Silver nitrate ($AgNO_3$, 99.8%), copper(II) chloride ($CuCl_2$, 99.5%), thioacetamide (TAA, 98%), selenium powder (99.5%), and oleylamine (OLA, ≥ 70%) were acquired from Sigma Aldrich. Methanol, acetone, and chloroform were obtained from standard commercial suppliers. All reagents were utilized as received without additional purification. Zinc nitrate hexahydrate ($Zn(NO_3)_2\cdot 6H_2O$, 99.9%), methyl methacrylate (MMA), and glutaraldehyde (GLD, 50%) were sourced from Tianjin Chemical Plant Co., Ltd. in Tianjin, China. Citric acid, ethanol, hydrogen peroxide (H_2O_2), and glucose were procured from Beijing Chemical Plant Co. in Beijing, China. Tetraethoxysilane (TEOS) and sodium borohydride ($NaBH_4$) were purchased from Sinopharm Chemical Reagent Co. in Tianjin, China. Glucose oxidase (GOD) was acquired from Amresco Co., Ltd. in Tianjin, China. Chitosan (CS) was obtained from Solarbio Co., Ltd. in Tianjin, China. Leucine-rich glioma inactivated 1 (LGI1), anti-N-methyl-D-aspartate-receptor antibody (Anti-NMDAR), anti-contactin-associated protein like 2 antibody (Anti-CASPR2), and anti-glutamic acid decarboxylase 65 kDa isoform antibody(Anti-GAD65) and anti-Leucine-rich glioma inactivated 1 antibody (Anti-LGI1) were sourced from Beijing Boisynthese Biotechnology Co., Ltd. in Beijing, China. Bovine serum albumin (BSA, 96-99%) was purchased from Beijing DingGuo Biotechnology Company in Beijing, China. 1-Ethyl-3-(3-dimethylaminopropyl) carbodiimide (EDC) was acquired from Sigma-Aldrich in Steinheim, Germany. L-Cysteine was procured from Alfa Aesar Co. Ltd. in Beijing, China. Human serum samples were provided by the First Hospital of Jilin University. Phosphate-buffered saline (PBS) was prepared conventionally: CS, LGI1, BSA, and Anti-LGI1 solutions were adjusted to pH=7.2 (0.01 M). All chemicals were of analytical purity and used without further refinement. Aqueous solutions were made using deionized water (DI water, 18.25 MΩ/cm) from a purification system.

Apparatus

UV-visible transmittance spectra were acquired using a UV-3101PC scanning spectrophotometer (Shimadzu Instruments Co. Ltd., Shanghai, China) over the wavelength range of 200-1100 nm. Sample morphology was characterized via a JSM-7500F field-emission scanning electron microscope (SEM) (JEOL Instruments Co. Ltd., Shanghai, China). Fourier transform infrared (FTIR) absorption spectra were recorded using a Shimadzu DT-40 model 883 infrared spectrophotometer (Shimadzu Instruments Co. Ltd., Shanghai, China). X-ray diffractometry (XRD) analysis was performed using a RigakuD/max 2550 X-ray diffractometer (Tiger Instruments Co. Ltd., Wuhan, China). Electrochemical impedance spectroscopy (EIS) measurements were conducted with a CHI660D electrochemical analyzer (ChenHua Instruments Co. Ltd., Shanghai, China). Electrochemical tests were carried out using a CHI630D electrochemical analyzer (ChenHua Instruments Co., Ltd., Shanghai, China) with a conventional three-electrode configuration: platinum wire as the counter electrode, saturated calomel electrode (SCE) as the reference electrode, and a modified electrode with a geometric area of 1.0 ± 0.1 cm^2 as the working electrode. Cyclic voltammetry (CV) curves were recorded at a scan rate of 100 mV/s. All photocurrent measurements were performed undera 500 W xenon lamp irradiationat a constant potential of 0.6 V (SCE) in 0.1 M PBS buffer (pH = 7.2).

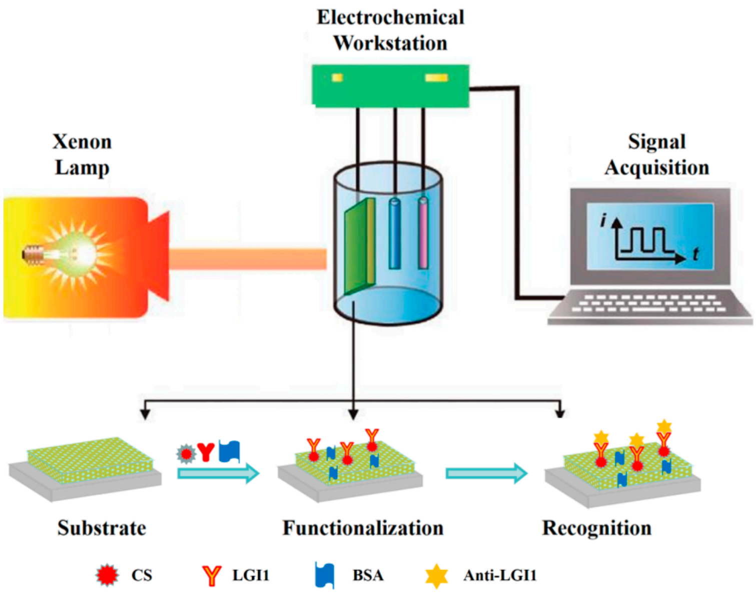

Scheme S1: The photoelectrochemical measurement process.

Table S1: Detection of Anti-LGI1 in human standard serum.

Added (ng/mL)	Measured (ng/mL)	RSD (%, n=5)	Recovery (%)
0	1.42	5.56	-
1	2.38	4.39	96.1

10	12.17	5.01	99.5
50	52.38	5.15	104.2
100	103.16	4.77	101.8

Table S2. Performance comparison of commercial anti-LGI1 or LGI1 detection methods and the developed PEC biosensor

Detection Method	Limit of Detection (LOD) or Sensitivity	Operation Duration	Reagent Cost per Test
Commercial ELISA Kit A (CUSABIO CSB-EL012898HU)	39 pg/mL, for LGI1	4.5 h	$ 6.25
Commercial ELISA Kit B (MyBioSource MBS904664)	39 pg/mL, for LGI1	4 h	$ 12.29
Cell-based Immunofluorescence Assay	10 pg/mL, for anti-LGI1 IgG	24 h	$ 41.35
This work (ZnO/AgCuS PEC biosensor)	13 pg/mL, for anti-LGI1 antibody	2.5 h	$ 3.8

Note: The reagent costs were estimated based on publicly available product prices and the number of assays provided per kit. For commercial ELISA kits, prices may vary depending on region, distributor, and purchasing quantity. For the PEC biosensor, the cost was calculated based on the consumption of electrode substrates, nanomaterials, antibodies, and other biochemical reagents used for a single assay.

Sources:

1.https://www.cusabio.com/ELISA-Kit/Human-Leucine-rich-glioma-inactivated-protein-1LGI1-ELISA-kit-87429.html#a03

2.https://www.mybiosource.com/lgi1-human-elisa-kits/leucine-rich-glioma-inactivated-1/904664

3.http://bjtjcni.com/laboratory/

Table S3. Synthesis parameter gradient screening results and optimized values

Parameter	Screening Range	Optimized Value	Performance at Optimized Value
ZnO calcination temperature	350-550°C	450°C	Maximum photocurrent response (12.8 μA), complete inverse opal structure retention
AgCuS precursor concentration	0.1-0.5 mmol	0.3 mmol	Uniform NC distribution, 6-fold photocurrent enhancement compared to pure ZnO
AgCuS reaction time	2-10 min	5 min	Narrow particle size distribution (12-18 nm), optimal energy level matching
